# The complete plastome sequence of *Tanakaea radicans* (Saxifragaceae) and its phylogenetic analysis

**DOI:** 10.1080/23802359.2021.1889409

**Published:** 2021-03-16

**Authors:** Ting Su, Guang-Ling Luo, Li Gu, Hai-Min Liao, Guo-Xiong Hu

**Affiliations:** a College of Life Sciences, Guizhou University, Guiyang, China; b The Key Laboratory of Plant Resources Conservation and Germplasm Innovation in Mountainous Region Ministry of Education, Guizhou University, Guiyang, China; c Institute of Agro-bioengineering, Guizhou University, Guiyang, China

**Keywords:** Complete chloroplast genome, Huecheroids, *Leptarrhena*, Saxifragaceae, *Tanakaea*

## Abstract

*Tanakaea radicans* is classified in the monotypic genus *Tanakaea* in the Saxifragaceae. It is a small, evergreen plant with a disjunct distribution in Japan and China. Here, we report and characterize for the first time the complete plastid genome sequence of *T. radicans*. The chloroplast genome is 155,265 bp in length and contains a pair of inverted repeats (25,794 bp) separated by a large single copy (86,289 bp) and a small single copy (17,388 bp). A total of 113 unique genes, including 79 protein-coding, 30 tRNA, and four rRNA genes, were annotated. Phylogenetic analysis showed that *T. radicans* was sister to *Leptarrhena pyrolifolia* within Huecheroids linage of Saxifragaceae.

The genus *Tanakaea* Franch. & Sav is a monotypic genus of Saxifragaceae (Pan and Soltis 2001). The current distribution of *T. radicans* Franch. et Sav. is disjunct, growing in Southwest China and Japan (Wang 1989; Pan and Soltis 2001; Sakaguchi et al. 2018). Morphologically, *Tanakaea* is similar to *Leptarrhena* R.Br., and phylogenetically, they together form a sister group within the Huecheroids lineage of Saxifragaceae (Soltis et al. 2001; Xiang et al. 2012; Deng et al. 2015). To date, the plastid genome of this monotypic genus has not been reported. In this study, we present the complete plastome sequence of *T. radicans* to contribute to the systematics and phylogenetic study of the Saxifragaceae.

The sample of *Tanakaea radicans* was collected from Huangnidong (107°25′12.73″, 29°3′39.53″, elevation 654 m), Xiangshi, Daqian, Daozhen, Zunyi, Guizhou, Southwest China, and specimens were deposited in the herbarium of the Natural Museum of Guizhou University (voucher: Hu et al. 658, GACP). Total genomic DNA was isolated from fresh leaves using a modified CTAB method (Doyle and Doyle 1987). Paired-end (PE) sequencing of 150 bp was conducted on an Illumina Hiseq-2500 platform at BGI-Wuhan. Approximately, 3 GB of raw short sequence data was generated and has been deposited in Sequence Read Archive (SRA) under accession number SRR13197184. The plastid genome was assembled *de novo* using the GetOrganelle script (Jin et al. 2020) and annotated with PGA (Qu et al. 2019) with *Leptarrhena pyrolifolia* (D.Don) Ser (MN496070) as the reference. The tRNA genes were further verified using the online tRNAscan-SE search servers with default parameters (Lowe and Chan 2016) and then manually adjusted in Geneious 10.0.5 (Kearse et al. 2012). Finally, the annotated genomic sequence of *T. radicans* was submitted to GenBank under the accession number MW300581.

The cp genome of *Tanakaea radicans* has a typical quadripartite structure (155,265 bp), including a pair of inverted repeats (IRa and IRb: 25,794 bp) separated by a large single copy (LSC: 86,289 bp) and a small single copy (SSC: 17,388 bp). The overall GC content is 37.91%. The GC content within LSC, SSC, and IR regions is 35.87%, 32.39%, and 43.20%, respectively. A total of 113 unique genes are successfully annotated, including 79 protein-coding, 30 tRNA, and four rRNA genes, of which 19 genes are duplicated in the IR regions. Among them, eight protein-coding genes (*atpF*, *ndhA*, *ndhB*, *petB*, *petD*, *rpl16*, *rpoC1*, and *rps1*6) and six tRNA genes (*trnA^-UGC^*, *trnG^-GCC^*, *trnI^-GAU^*, *trnK^-UUU^*, *trnL^-UAA^*, and *trnV^-UAC^*) contain one intron, and three genes (*clpP*, *rps12*, and *ycf3*) included two introns.

To validate the phylogenetic position of *Tanakaea radicans* within the Saxifragaceae, a phylogenetic analysis was carried out by analyzing the cp genomes of *T. radicans* with 17 other species (including 16 ingroups within the Saxifragaceae and two outgroups from Grossulariaceae and Iteaceae). Sequences were aligned using MAFFT 7.409 (Katoh and Standley 2013), and a maximum-likelihood (ML) analysis was conducted using RAxML-HPC2 on XSEDE v.8.1.11 (Stamatakis 2014) as implemented on the CIPRES computer cluster (http://www.phylo.org/) (Miller et al. 2010) with *Itea chinensis* Hook. & Arn (MH191391) and *Ribes roezlii* Regel (MN496076) as outgroups. With the exception of the bootstrap replicates set at 1000, the remaining parameters followed default. The ML tree revealed that *Tanakaea* is sister to *Leptarrhena* within Huecheroids lineage of Saxifragaceae (Figure 1). Phylogenetic position of *Tanakaea* inferred by cp genomic sequences is the same as previous studies (Soltis et al. 2001; Xiang et al. 2012; Deng et al. 2015).

**Figure 1.**
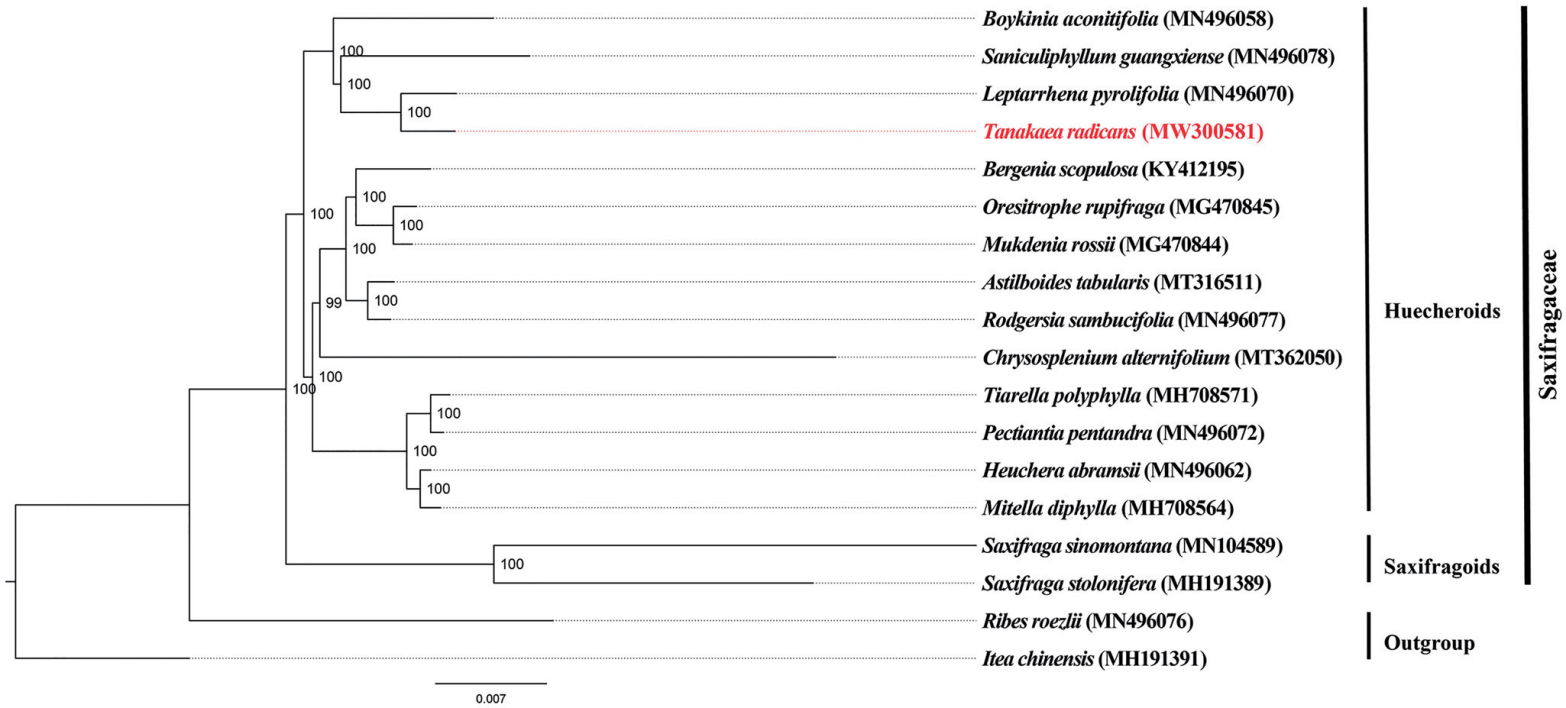
The maximum-likelihood tree of Saxifragaceae inferred from 18 complete chloroplast genomes with *Ribes roezlii* and *Itea chinensis* as outgroups. The position of *Tanakaea radicans* is highlighted in bold.

## Data Availability

The genome sequence data supporting this study are openly available in GenBank nucleotide database, https://www.ncbi.nlm.nih.gov/nuccore/MW300581, Associated BioProject, https://www.ncbi.nlm.nih.gov/bioproject/PRJNA682524, BioSample accession number at https://www.ncbi.nlm.nih.gov/biosample/SAMN16992795, and Sequence Read Archive at https://www.ncbi.nlm.nih.gov/sra/SRR13197184.
